# Reassessing the Baveno based strategy in China: a cost-effectiveness analysis of screening for high-risk varices in cirrhosis

**DOI:** 10.3389/fpubh.2026.1779291

**Published:** 2026-03-06

**Authors:** Shuhao Su, Jie Luo, Chongxiao Li, Caiyun Yang, Jiaqi Yang, Dawei Ding, Xingchen Liu, Guanya Guo, Ying Han

**Affiliations:** National Clinical Research Center for Digestive Diseases, Xijing Hospital of Digestive Diseases, The Air Force Military Medical University, Xi'an, China

**Keywords:** cirrhosis, detection, economic evaluation, high-risk varices, markov model

## Abstract

**Background & Aims:**

The Baveno consensus recommends using liver stiffness measurement (LSM) and platelet count to avoid endoscopy in low-risk patients with compensated advanced chronic liver disease (cACLD). This study aimed to compare the cost-effectiveness of the Baveno-based selective screening vs. universal screening strategy for high-risk varices (HRV) in a Chinese cACLD cohort.

**Methods:**

A state-transition Markov model was constructed from the Chinese healthcare system perspective, simulating a cohort of 1,000 patients with cACLD over a five-year horizon. Model inputs were derived from Chinese real-world data, meta-analyses, and national fee schedules. Outcomes included costs, quality-adjusted life years (QALYs), incremental cost-effectiveness ratio (ICER), bleeding events, and endoscopic volumes. Sensitivity analyses and scenario analyses were performed to assess the uncertainty.

**Results:**

The selective screening strategy yielded additional QALYs gain (3.4780 vs. 3.4452) and higher cost ($581 vs. $512) over 5 years compared with universal screening, resulting in an incremental cost of $2,103.66 per additional QALY gained and sparing 26% of patients from initial endoscopy. This ICER falls below China's per-capita GDP-based willingness-to-pay (WTP) threshold, indicating that the selective screening is cost-effective in the Chinese context. Deterministic analysis supported these findings, and probabilistic sensitivity analysis showed that selective screening was the preferred strategy in the majority of simulations.

**Conclusion:**

Within China's healthcare context, the Baveno-based selective screening strategy is a cost-effective option for screening of high-risk varices in patients with cACLD, suggesting that it should be considered for widespread implementation on clinical and economic grounds.

## Introduction

Cirrhosis is characterized by the fibrotic replacement of hepatic tissue, arising from various chronic liver conditions. A major progressive complication is portal hypertension, which can lead to severe outcomes such as ascites, hepatic encephalopathy, and variceal bleeding (1, 2). Among these, variceal hemorrhage is a life-threatening event, occurring in 25–40% of patients with cirrhosis and carrying mortality rate of up to 20% (3). The severity of esophageal varices is closely associated with the degree of liver dysfunction (4), and after an initial bleeding event, the one-year recurrence risk is as high as 50% (5). Given this high burden, the prevention of the first bleeding event through timely screening is a cornerstone of management.

The clinical paradigm has evolved with the introduction of the term “compensated advanced chronic liver disease” (cACLD), which describes a continuous spectrum from significant fibrosis to compensated cirrhosis in patients with progressive chronic liver disease (6). This concept offers greater clinical utility than the traditional dichotomous classification. Patients with cACLD are at elevated risk of decompensating events, with esophagogastric variceal hemorrhage being one of the most common and severe (7, 8).

The gold standard for detecting high-risk varices (HRV) remains conventional upper gastrointestinal endoscopy (9). However, its universal application as a screening tool is limited by considerable costs, patient discomfort, and inherent procedural risks. This has prompted the exploration of non-invasive diagnostics to develop personalized screening strategies, particularly through the identification of low-risk subgroups in whom endoscopy could be safely avoided. A prior cost-effectiveness analysis by Brennan et al. (10) suggested that a selective screening strategy based on non-invasive tests yielded only modest effectiveness gains at substantial cost, highlighting the need for more optimized approaches. In response, the Baveno VI consensus proposed a pragmatic solution: using liver stiffness measurement (< 20 kPa) and platelet count (>150,000/μL) to identify patients at very low risk of HRV and avoiding unnecessary endoscopy (6). These criteria have been validated and deemed cost-effective in several high-income countries (11, 12). However, the generalizability of this finding to other healthcare systems and etiologies of liver disease remains unclear, particularly in China. Chronic hepatitis B virus (HBV) infection is the predominant cause of cirrhosis in China, accounting for over 50% of cases (13). Although endoscopy is increasingly accessible and affordable (14), it is repeatedly applied in the long-term monitoring of a large HBV-related cACLD population. In turn, could accumulating substantial physical discomfort and financial burdens over time.

Therefore, this study conducts a comprehensive cost-effectiveness analysis comparing Baveno-based selective screening with universal screening for HRV in a Chinese cACLD cohort. By incorporating key local contextual factors, we aim to identify the optimal screening strategy and thus provide robust, locally relevant evidence to inform clinical guidelines and health policy decisions in China and similar settings.

## Materials and methods

A cost-effectiveness model was developed from the Chinese healthcare system perspective to compare the Baveno-based selective screening vs. universal screening for detecting HRV in patients with compensated advanced chronic liver disease. Although the Baveno VII consensus (2022) reaffirmed the Baveno VI criteria, recommending no endoscopy for patients with liver stiffness < 20 kPa and platelet count (PLT) > 150 × 10^9^/L (15). The universal endoscopy remains common in Chinese practice. The model simulated variceal natural history, screening, and treatment pathways to project long-term clinical and economic outcomes. Key parameters were sourced from real-world studies, meta-analyses, pilot programs, and official Chinese data.

### Model structure

**Figure 1:**
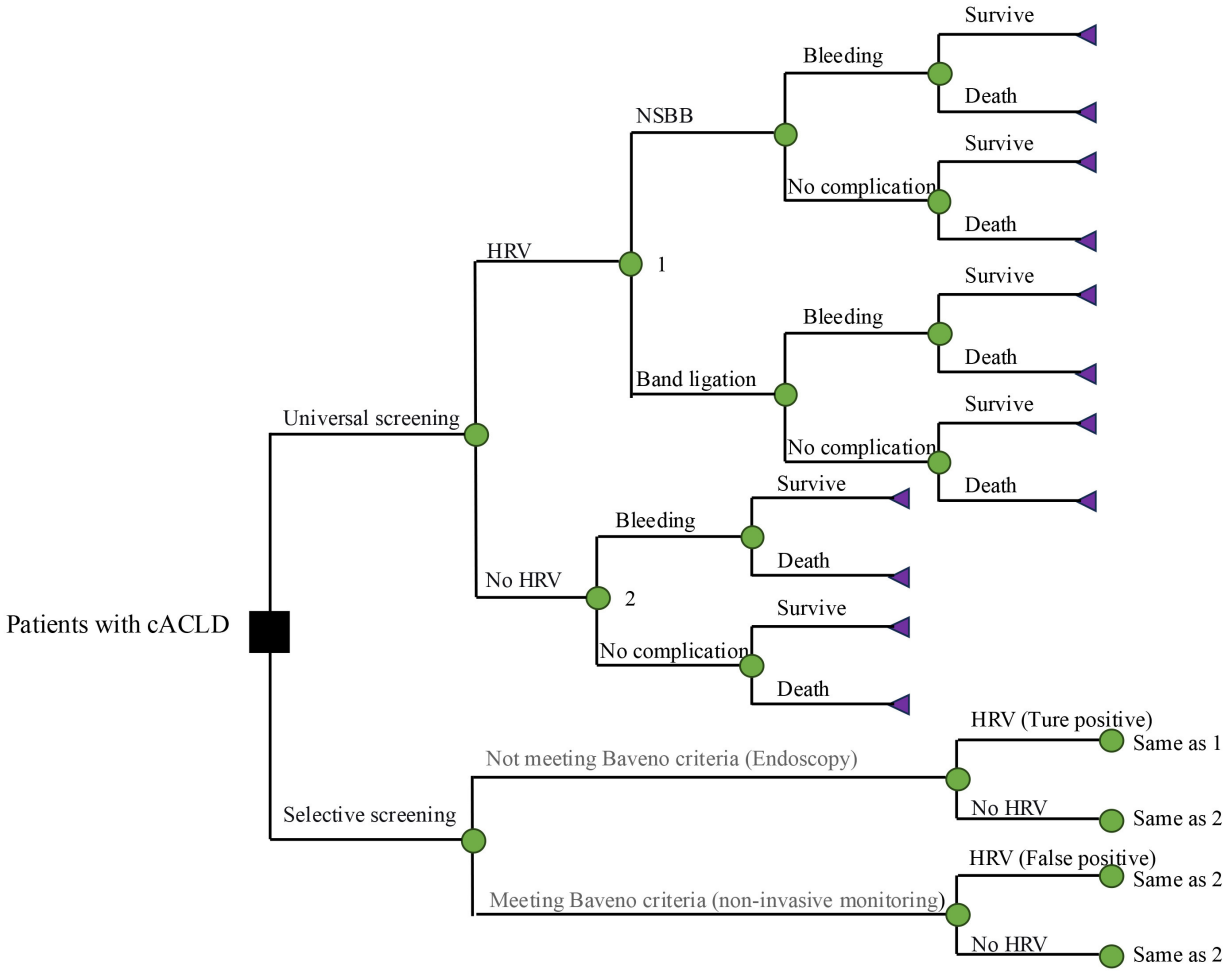
The decision tree model. A decision tree model was used to calculate first-year test outcomes and costs, and to distribute patients to initial health states in the Markov state transition model for the long-term simulation. In the universal screening strategy, all patients undergo initial endoscopy. In the selective strategy, patients are first stratified by the Baveno VI criteria; those who meet the criteria enter annual non-invasive monitoring, while others proceed directly to endoscopy. Patients diagnosed with HRV receive NSBBs or endoscopic band ligation. cACLD, compensated advanced chronic liver disease; HRV, high-risk varices; NSBBs, non-selective beta-blockers.

An individual-level state-transition Markov model was developed in TreeAge Pro Healthcare 2021 to evaluate the clinical and economic impacts of selective vs. universal screening for HRV in patients with cACLD (Figure 1). The base case cohort simulated 1,000 patients diagnosed with compensated cirrhosis and suspected esophagogastric varices, with no history of decompensation events (e.g., ascites, variceal bleeding, or hepatic encephalopathy). Clinical management followed national and international guidelines. Patients with contraindications to non-selective beta-blockers (NSBBs) or endoscopic band ligation were excluded.

The model employed a one-year cycle length and a five-year horizon. In the selective screening strategy, patients meeting Baveno criteria underwent annual non-invasive monitoring without initial endoscopy. All other patients proceeded to endoscopy, and those confirmed with HRV initiated NSBBs or ligation. In contrast, the universal screening strategy mandated initial endoscopy for all patients. Those without HRV entered a follow-up program with repeat endoscopy every two years. To maintain the integrity of the model, we incorporated transitions for variceal bleeding and death from all causes, although the incidence rate was very low.

### Modeling assumptions

Several key assumptions were incorporated into the model due to data limitations and for computational tractability. The simulated cohort consisted of treatment naive patients, with no prior history of variceal screening or diagnosis. Each patient could experience at most one bleeding event, and that the risk of bleeding was independent of the risk of non-liver-related death. To focus the analysis on the natural history of varices, other decompensation events, progression to hepatic dysfunction, and the development of hepatocellular carcinoma were excluded. For the simplicity of the model, patients on NSBBs continued therapy throughout the model's time horizon, undergoing endoscopy only after a bleeding event; patients receiving ligation underwent repeat procedures biennially with annual endoscopy. Finally, the model accounted for the false-negative rate of the Baveno VI criteria to reflect the risk of missing HRV.

### Model inputs

Input parameters were sourced from real-world studies, meta-analyses, pilot programs, and official Chinese databases, including variceal prevalence, disease progression, bleeding probability, mortality, and transition probabilities (Table 1) (16–26). Sensitivity and specificity of Baveno criteria were derived from a published meta-analysis (11). Given the pre-dominance of HBV in China (26), which has a different clinical course than alcohol-related or Hepatitis C virus (HCV)-related cirrhosis in Western populations, the prevalence of HRV was estimated from multiple high-quality domestic clinical studies conducted in China. Mortality from chronic hepatitis B (CHB) was set equal to the all-cause mortality of the general Chinese population (27, 28).

**Table 1:** Transition probabilities.

**Parameters**	**Value**	**Range**	**References**
Prevalence of HRV	0.20	0.10–0.30	16–19
Meeting Baveno criteria	0.26	0.17–0.40	Calculates based on the prevalence of HRV, sensitivity, and specificity values
Sensitivity	0.97	0.95–0.98	11
Specificity	0.32	0.26–0.39	11
PPV	0.26	0.22–0.36	Calculates based on the revalence of HRV, sensitivity, and specificity values
NPV	0.98	0.93–0.99	Calculates based on the revalence of HRV, sensitivity, and specificity values
Probability of NSBBs treatment	0.8	0–1	20, 21
Probability of band ligation treatment	0.2	0–1	20, 21
Bleeding after missed HRV	0.10	0.08–0.12	22
Bleeding after NSBBs	0.068	0–0.1	23
Bleeding after band ligation	0.043	0–0.1	24
Death after bleeding	0.022	0.01–0.04	25
Death due to other reasons	0.034	0–1	26

HRV, high-risk varices; PPV, positive predictive rate; NPV, negative predictive rate; NSBBs, non-selective beta-blockers.

Costs data were sourced from the Chinese national fee schedules and hospital electronic medical record, incorporating direct medical expenses only (Table 2) (29–33). The annual cost of NSBBs was estimated at $52.68 (26.34–79.02), based on a standard treatment with 12.5 mg/d of carvedilol. Half of patients were assumed to choose painless endoscopy, with cost adjusted accordingly. The cost of managing a bleeding episode encompassed all direct inpatient and outpatient care. All costs were converted to 2022 USD ($1 = ¥6.7261). Health utilities were sourced from published studies. Both costs and utilities were discounted at an annual rate of 3.5%.

**Table 2:** Costs and utilities.

**Parameters**	**Value**	**Range**	**Distribution**	**References**
**Costs**				
Endoscopy	$96.73	$44.64–$178.57	Gamma	EMR
Blood routine	$2.68	$2.23–$3.72	Gamma	EMR
Elastography	$18.60	$12.65–$24.55	Gamma	EMR
NSBBs, 1 year	$52.68	$26.34–$79.02	Gamma	EMR
Band ligation	$744.05	$446.43–$1,026.79	Gamma	EMR
Variceal bleeding, hospitalization	$1,934.52	$758.93–$2,976.19	Gamma	29
**Utilities**				
Compensated cirrhosis, NSBBs	0.65	0.52–0.95	Beta	30,31
Compensated cirrhosis, band ligation	0.67	0.52–0.95	Beta	30,31
Negative for HRV	0.76	0.52–0.95	Beta	32
People with HRV not treated	0.72	0.75–0.90	Beta	32
Variceal bleeding	0.54	0.26–0.66	Beta	31,33

HRV, high-risk varices; EMR, electronic medical record; NSBBs, non-selective beta-blockers.

In scenario analysis, the Baveno VII consensus has redefined the management of cACLD by emphasizing the treatment of all patients with clinically significant portal hypertension (CSPH) to prevent any form of decompensation, moving beyond the sole prevention of variceal bleeding in a HRV subgroup. As a cornerstone of this strategy, CSPH could be ruled in with a LSM ≥ 25 kPa with high specificity and positive predictive value (34). In this scenario, patients with LSM ≥ 25 kPa are initiated on NSBBs without undergoing endoscopy. Only the remaining patients in the diagnostic “gray zone” (LSM < 25 kPa) undergo endoscopic screening for HRV, as they do not meet the definitive criteria for CSPH and still require risk stratification for varices.

### Statistical analysis

The incremental cost-effectiveness ratio (ICER) was evaluated against with willingness-to-pay (WTP) thresholds, set at China's 2022 per capita gross domestic product (GDP) ($12,714.11 per QALY). The ICER was applied in both base-case analysis and probabilistic sensitivity analysis (PSA). To assess the impact of parameter uncertainty on model outcomes, comprehensive sensitivity analyses were performed. Parameter ranges were defined using 95% confidence intervals where available. For costs associated with transient elastography (TE), PLT, and endoscopy, upper and lower limits were based on the highest and lowest documented medical service prices in Shanghai, Guangzhou, and Zhejiang. For all other parameters, a variation range of ±20% was applied. We further examined the influence of HRV prevalence and variations in endoscopy cost on model conclusions. PSA was conducted using Monte Carlo simulation with 10,000 iterations, employing beta distributions for probabilities and utilities, gamma distributions for cost parameters, and triangular distributions where specified.

## Results

### Screening results & costs of different screening strategies

Application of the Baveno criteria in the selective screening strategy spared 26% of patients from initial endoscopy. In a simulated five-year model of 1,000 patients, the universal screening strategy resulted in 80 bleeding events, compared with 90 events under the selective strategy. By performing endoscopy on all patients, universal screening strategy correctly identified all 200 patients with HRV for primary prophylaxis (NSBBs or ligation). In contrast, the selective strategy yielded false-negative results, missing an estimated 6.8 HRV cases per 1,000 patients.

**Figure 2:**
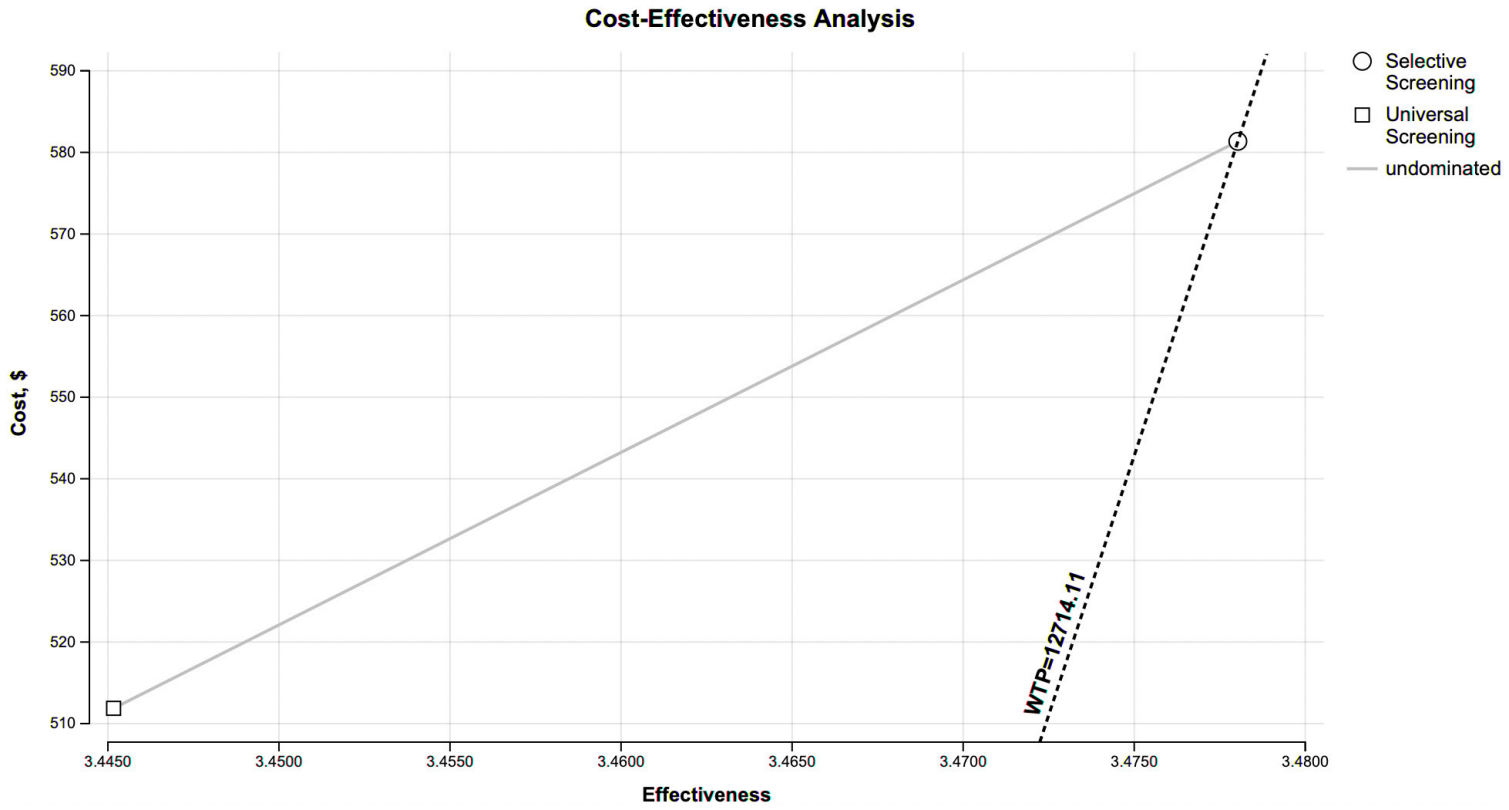
Cost effectiveness analysis shows that selective screening strategy has a higher cost than universal screening ($581 vs. $512) and greater effectiveness (3.4780 vs. 3.4452 QALYs). At the willingness to pay threshold of one time China's per-capita GDP ($12,714.11), the selective screening strategy is a cost-effective option. WTP, willingness-to-pay; QALY, quality-adjusted life years.

The base-case results are summarized in Table 3. Selective screening was associated with an incremental gain of 0.0328 QALYs per person compared with universal screening, and this QALY gain was achieved at a higher per-person cost ($581 vs. $512). The resulting ICER for the selective strategy was $2,103.66 per QALY gained relative to universal screening. This ICER falls below the WTP threshold of one time China's per-capita GDP (Figure 2), indicating that the selective strategy meets the criterion for being cost-effective in the Chinese context.

**Table 3:** Base case results.

**Strategy**	**Average cost per person tested**	**Incremental cost**	**Effectiveness**	**Incremental effectiveness**	**ICER**
Universal screening	$512	–	3.4452	–	–
Selective screening	$581	$69	3.4780	0.0328	$2,103.66

ICER, incremental cost-effective ratio.

### Sensitivity analysis

#### Deterministic sensitivity analysis

One-way sensitivity analysis identified endoscopy cost as a critical determinant of cost-effectiveness. Given the widespread availability and affordability of non-anesthetic endoscopy in China, we evaluated the influence of its cost on preference of strategy. The model demonstrated that universal screening remained less costly over the five-year time horizon as long as the cost per endoscopy was below $192.59 (Supplementary Figure S1). However, once the endoscopy cost exceeded this threshold, the selective strategy became the dominant option, being both less expensive and more effective. Beyond this price point, the NMB advantage increasingly favored the selective strategy with rising endoscopic prices. The analysis also tested the robustness of the findings to variations in the prevalence of HRV. When the prevalence was less than 42%, the model results remained consistent, with the selective screening strategy continuing to demonstrate favorable cost-effectiveness compared to universal screening.

Based on the Tornado diagram (Supplementary Figure S2), several key parameters were identified as major drivers of uncertainty. The prevalence of HRV and the cost of endoscopy exerted the strongest influence on the ICER, confirming their critical role in determining the economic outcomes of screening strategies. Furthermore, the cost of post-bleeding hospitalization and the cost of non-invasive assessments (including transient elastography and platelet count) also substantially impacted the results. Additionally, the utility value assigned to health states after bleeding events significantly affected ICER estimates, underscoring the importance of quality-of-life measurements in the model. These findings highlight that both clinical parameters and cost-related factors are significant sources of uncertainty in evaluating the cost-effectiveness of screening strategies for HRV.

A two-way sensitivity analysis was performed to evaluate the impact of varying the sensitivity and specificity of the Baveno criteria. At a WTP threshold of one time China's per-capita GDP, selective screening would become cost-effective only if the Baveno criteria achieved a sensitivity of at least 0.90 and specificity of at least 0.25 simultaneously.

#### Probabilistic sensitivity analysis

**Figure 3:**
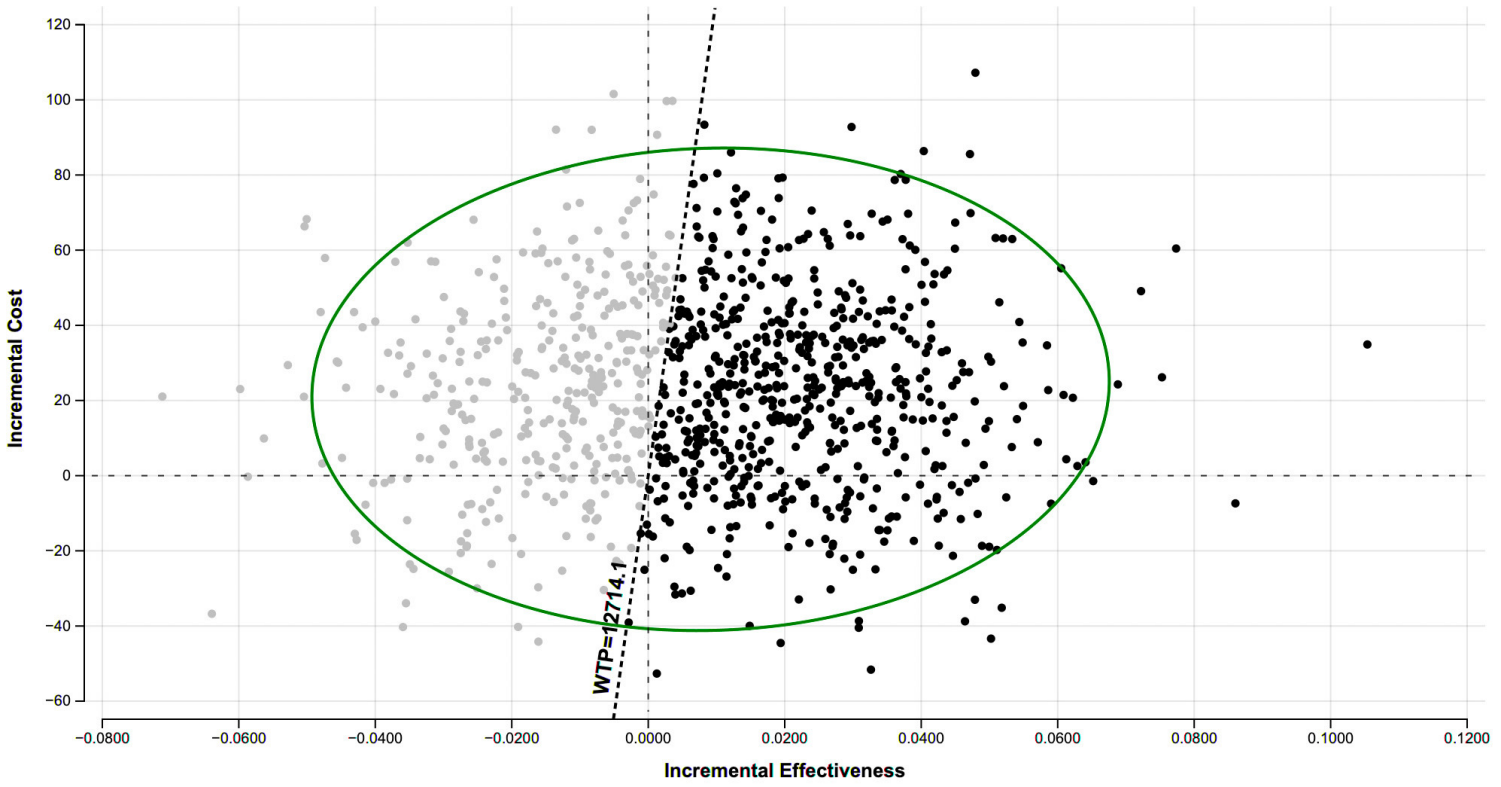
Probabilistic sensitivity analyses (PSA) results are shown in scatter plot of incremental cost effectiveness ratio. The black dots represent where selective screening is the more cost-effective option at one time China's per-capita gross domestic product (GDP) ($12,714.11) for a range of increases in incremental effectiveness values. WTP, willingness-to-pay.

To account for parameter uncertainty, we performed a probabilistic sensitivity analysis (PSA) using 10,000 Monte Carlo simulations, sampling all parameters from their respective probability distributions. The resulting scatter plot on the cost-effectiveness plane (Figure 3) showed that the probability of selective screening being cost-effective was 57.2% at a WTP threshold equivalent to one time China's per-capita GDP. The cost-effectiveness acceptability curve (CEAC) further supported this finding (Supplementary Figure S3). Selective screening was the preferred strategy in the majority of simulations. Below a threshold of $4,322, universal screening was more likely to be cost-effective, reflecting its economic advantage when cost containment is prioritized. Above this threshold, selective screening consistently demonstrated a higher probability of being cost-effective.

### Scenario analysis

To explore an alternative management strategy aligned with Baveno VII consensus, we simulated a strategy in which patients with a LSM ≥ 25 kPa directly initiated NSBBs without undergoing endoscopy, while only those with LSM < 25 kPa undergo endoscopic screening for HRV. This scenario analysis demonstrated that compared to the base case universal screening strategy, this new strategy saved a greater number of endoscopies and yielded a lower five-year cost per patient ($511 per patient). However, the increased use of NSBBs resulted in a slight reduction in effectiveness, with an average gain of 3.41 vs. 3.44 QALYs for universal screening. One way sensitivity analysis further identified that for the strategy to become cost effective, the prevalence of HRV among patients with CSPH would need to exceed 50%.

## Discussion

This economic evaluation compared the Baveno-based selective screening with the universal screening strategy for HRV in a Chinese cACLD population. The base-case analysis demonstrated that the selective screening spared 26% of patients from initial endoscopy and yielded an additional QALY gain of 0.0328 per patient, but at a higher per-person cost ($581 vs. $512). The resulting ICER of $2,103.66 per QALY gained falls below China's per-capita GDP-based WTP threshold, indicating that the selective strategy meets the criteria for being cost-effective in the Chinese context. Importantly, the selective screening remained cost-effective in sensitivity analyses. This study assessed the cost-effectiveness of these criteria and makes the argument for more widespread implementation.

The Baveno VI criteria have a very high specificity, thus minimizing the proportion of patients with false-negative results. Even if HRV are missed, retesting happens within 12 months, thus reducing the probability of variceal bleeding. In China, approximately 74% of patients with cirrhosis undergo endoscopic screening, but the majority were found to have no HRV (35). The false-negative rate translates into missed HRV diagnoses and missed opportunities for primary prophylaxis, leading to bleeding events and associated costs (36). It is acknowledged that endoscopy, as an invasive procedure, carries inherent risks of complications such as bleeding, perforation, or infection, especially when examinations are repeated multiple times.

Furthermore, the high prevalence of HBV dominates the etiology of cirrhosis in China, contributing to a prolonged and distinct natural history of portal hypertension and variceal development compared to Western populations with HCV or alcohol-related liver disease. This context increases the clinical value of screening for HRV. For patients with HBV-related cACLD, who typically require lifelong monitoring, the prospect of repeated endoscopy procedures over decades can be a source of significant psychological burden. The selective strategy also reduces anxiety for patients and the discomfort of an invasive procedure.

The low cost of endoscopy in China is a key factor influencing our results. Sensitivity analysis confirmed that universal screening remains cost-saving when the endoscopy cost is below $192.59. Given that standard endoscopy often costs between $45 and $75 (RMB ¥300–500), the economic incentive to avoid the procedure via non-invasive triage is substantially diminished (14). Although some studies suggest that non-invasive screening for varices in cirrhosis patients yields a similarly low risk of future bleeding compared to endoscopy (37), the limited sample sizes and the objectively higher bleeding risk in missed HRV patients who remain untreated must be considered in model development. From a clinical perspective, the avoidance of bleeding events represents a meaningful advantage for universal screening, which perhaps indicates endoscopy cannot be completely replaced by non-invasive methods.

We also explored a scenario informed by the Baveno VII consensus, where patients with LSM ≥ 25 kPa initiate NSBBs without initial endoscopy. While this approach saved more endoscopies, it was associated with a slight reduction in QALYs, as 5 to 10% of patients may be misclassified and receive NSBBs in the absence of definitive CSPH, potentially exposing them to side effects that reduce quality of life without compensatory benefit (38). It is crucial to acknowledge that NSBBs treatment in patients with CSPH aims to prevent not only variceal bleeding but also other decompensation events. Consequently, a more comprehensive modeling framework that fully incorporates the preventive effect of NSBBs against all forms of decompensation would likely demonstrate greater clinical and economic advantages for non-invasive, CSPH-targeted strategies. The development of such a model represents an important and necessary direction for future cost-effectiveness research, especially as non-invasive tools for diagnosing and monitoring CSPH continue to evolve in accuracy and accessibility.

Furthermore, our model did not take into account other benefits of gastroscopy, including the early detection of concurrent upper gastrointestinal malignancies, which share common risk factors with liver disease and are prevalent in China (39). A large multicenter cohort study in China (*n* = 637,500) demonstrated reductions of 23% in incidence and 57% in mortality for upper gastrointestinal cancers among screened individuals compared to controls (40). Prognosis is highly stage-dependent, with five-year survival exceeding 85% for early-stage lesions but dropping below 10% in advanced disease (41). Individualized risk assessment and regular endoscopic surveillance thus play essential roles in the prevention and early management of digestive tract diseases.

Diagnosis-related groups (DRGs) represent an advanced payment system that classifies patients into groups based on diagnosis, severity, and complications to standardize management and reimbursement. More recently, nations including China, Russia, Thailand, and South Korea have begun adapting DRG systems to local contexts, with the goal of optimizing resource allocation, improving service delivery, and increasing patient satisfaction (42, 43). In China's evolving DRG framework, the selective screening strategy for patients with cirrhosis is viable at the current WTP threshold. Annual screening aligns well with DRG objectives by ensuring timely detection of varices without significantly escalating costs (44). Thus, within a value-based reimbursement context, selective screening supports both clinical comprehensiveness and economic sustainability.

In future clinical practice, upper endoscopy may still not be completely replaced, but rather complement non-invasive methods in dynamic risk stratification and monitoring. All patients with cACLD undergo initial upper endoscopy to definitively establish the presence and grade of varices. Following initial screening, surveillance intervals can be individualized: patients found to have no varices or low-risk varices, and who concurrently meet Baveno criteria, represent a very low-risk subgroup. They may be considered for extended surveillance intervals (e.g., every 2–3 years), guided by serial non-invasive tests; Patients not meeting these criteria, or those with high-risk varices on primary prophylaxis, should continue regular endoscopic surveillance as per guidelines (e.g., every 1–2 years). For the majority with HBV etiology, effective antiviral therapy may be integrated into the model. Regular LSM monitoring can capture regression of liver stiffness. A significant and sustained LSM decline (e.g., to < 15 kPa) could prompt re-evaluation and potential lengthening of surveillance intervals in consultation with endoscopic findings, creating a truly dynamic management loop (45). This “universal baseline diagnosis plus non-invasive surveillance” strategy leverages the affordability of endoscopy in China to secure an unambiguous initial diagnosis, while employing non-invasive tools efficiently to personalize long-term management frequency. It balances diagnostic thoroughness with resource rationality, aligning with both clinical imperatives and the economic realities of the Chinese healthcare system.

The study had some limitations. The first limitation of the model is the assumption that patients experience at most one variceal bleed over the five-year horizon, the risk of rebleeding is determined by portal hypertension severity and primary prophylaxis efficacy, not the screening strategy. The model focused on the screening and treatment of varices, did not incorporate other decompensation events or hepatocellular carcinoma, and it may not fully capture the intricacies of disease management in patients with cirrhosis. We only calculated direct medical costs and did not include indirect and social costs in order to maintain the focus and feasibility of the study. Model parameters may not fully capture the heterogeneity of the cirrhotic population in China. As the model was constructed from the Chinese healthcare system perspective and relies on current local practices and pricing, its generalizability to other settings may be limited. Furthermore, evolving endoscopic technologies and shifts in clinical practice, such as increasing adoption of painless endoscopy, could influence future cost-effectiveness outcomes.

In conclusion, this analysis demonstrates that, within China's healthcare context, the selective strategy based on the Baveno criteria is a cost-effective option for screening high-risk varices in patients with cACLD. These evidence-based insights should inform the ongoing development of national clinical guidelines and health policy.

## Data Availability

The original contributions presented in the study are included in the article/Supplementary material, further inquiries can be directed to the corresponding authors.
