# Supplementary material for: Reassessing the Baveno based strategy in China: a cost-effectiveness analysis of screening for high-risk varices in cirrhosis
Source: Front Public Health. 2026 Mar 6;14:1779291. doi: 10.3389/fpubh.2026.1779291 (PMC13002583; doi:10.3389/fpubh.2026.1779291)
Supplement: Supplementary file 1 [file Data_Sheet_1.docx]

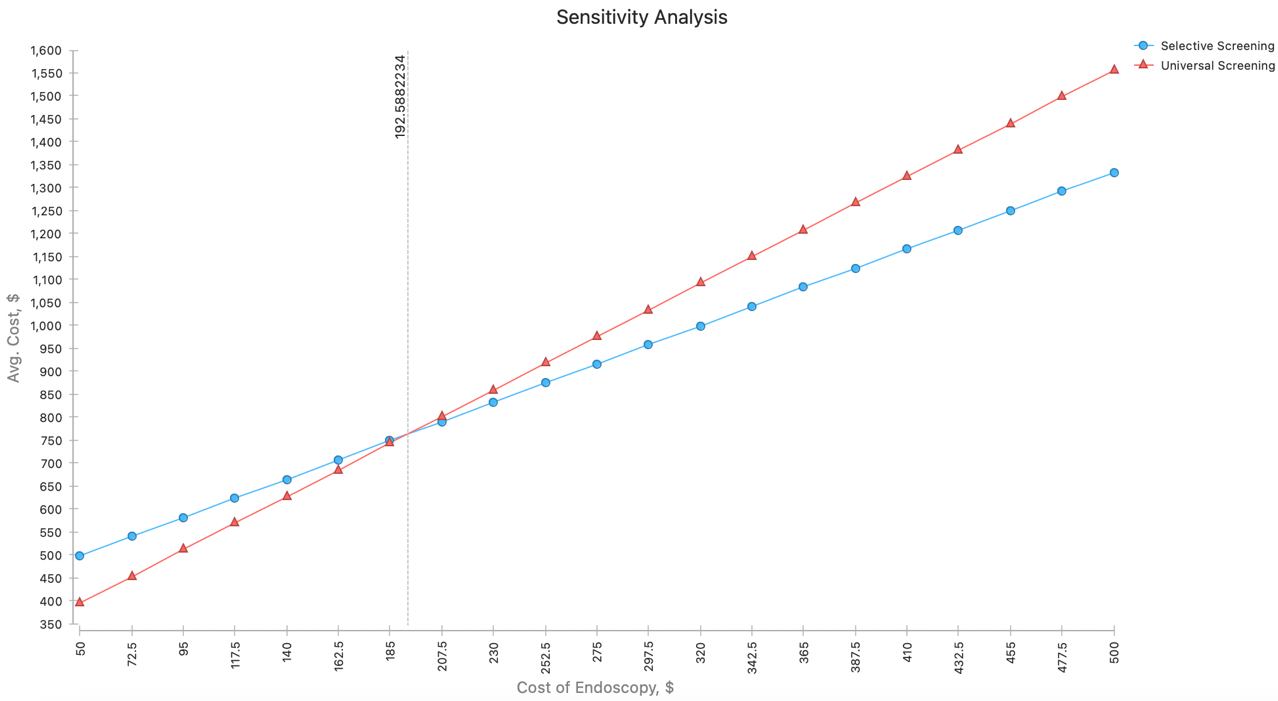


Figure S1. One-way sensitivity analysis for endoscopy cost. Universal screening remained cost-saving over a five-year horizon when endoscopy costs were below $192.59.


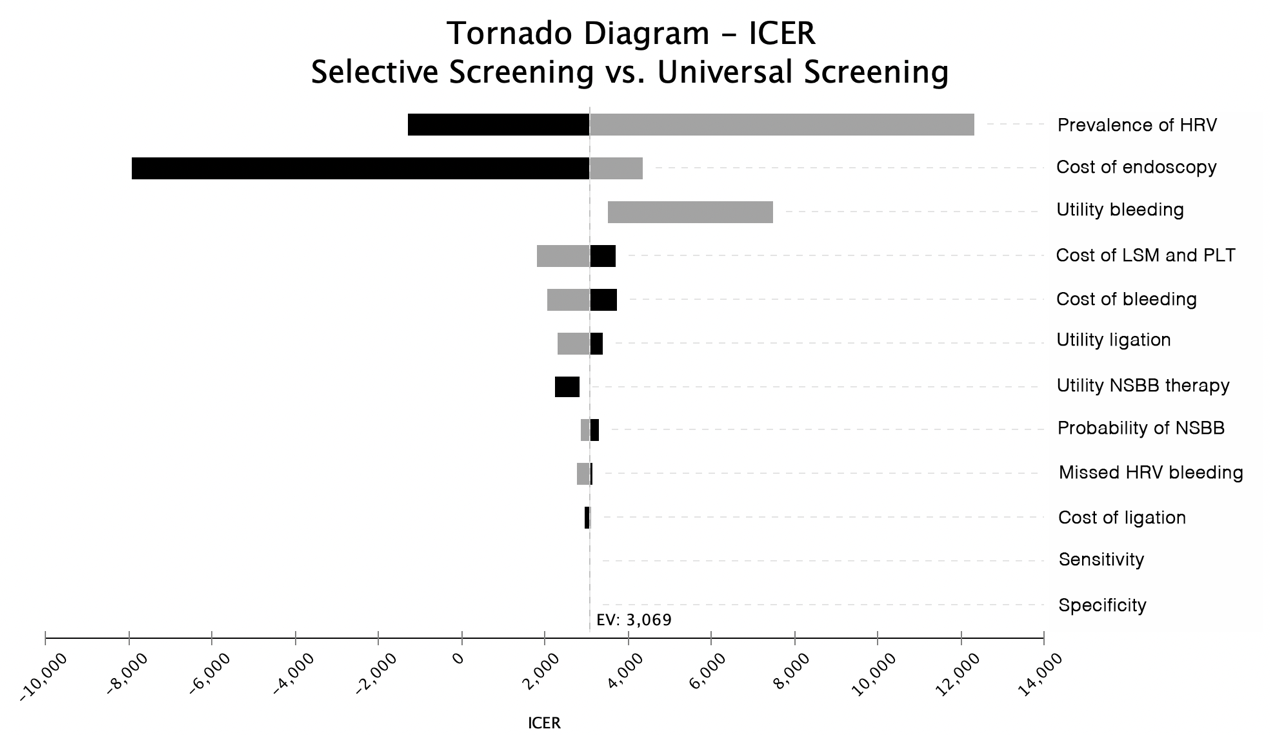


Figure S2. Tornado diagram of the incremental cost effectiveness ratios (ICER) for test characteristics, costs and utilities. This diagram represents the parameters that have the greatest impact with the width of the bar representing impact on model results. The specificity of both prevalence of HRV and the cost of endoscopy has the greatest impact on the model. Abbreviations: HRV, high-risk varices; NSBB, non-selective beta-blockers; EBL, endoscopic band ligation. ICER, incremental cost-effective ratio.


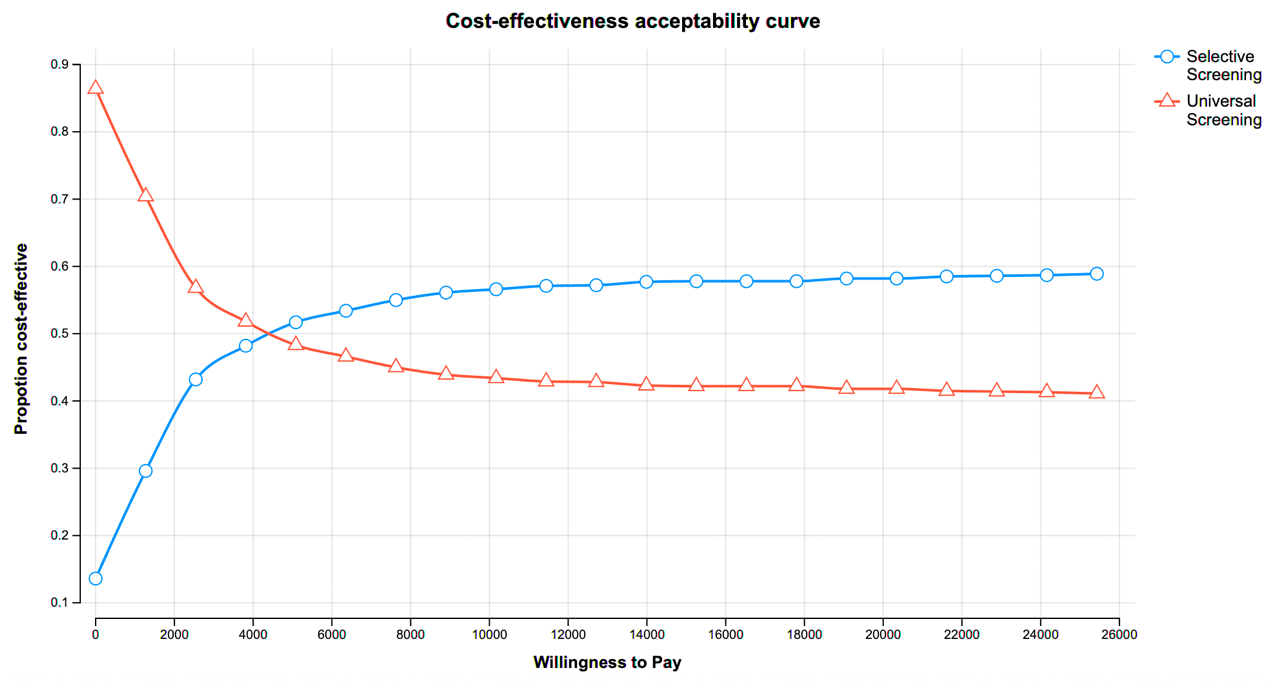


Figure S3. The cost-effectiveness acceptability curve. Above a willingness-to-pay threshold of $4,322, the selective screening had the higher probability of being cost-effective.
